# Research on the stability of support structures in steeply dipping coal seam working face

**DOI:** 10.1038/s41598-026-51153-2

**Published:** 2026-05-03

**Authors:** Yanhua Guo, Yanjie Zhang, Xian-gang Han, Teng-jiao Wang, Chi Zhu, Haoyu Song

**Affiliations:** School of Civil Engineering, Hebei University of Engineering, Handan，Hebei, 056038 P. R. China

**Keywords:** Steeply dipping coal seam, Anti-slip of the support, Anti-tilt of the support, Working resistance, Energy science and technology, Engineering

## Abstract

Stability control of overlying strata and support equipment in steeply inclined coal seam faces poses a significant challenge in mining operations. The implementation of curved face layouts has been proven effective in enhancing support stability. This study establishes mechanical models for both sliding and toppling instability of supports in steeply inclined faces. Critical stability equations are derived under optimal roof-contact conditions, and the minimum yield resistance required to maintain stable configurations in both inclined straight and curved face sections is determined. Theoretical analysis demonstrates that curved face geometries improve the stress distribution of lower-face supports. Field monitoring validate the practical efficacy of this approach. These findings provide valuable guidance for safe and efficient mining under similar geological conditions.

## Introduction

Steeply dipping coal seams, typically defined as having a dip angle greater than 35° in mining contexts^1^, account for approximately 10% to 20% of China’s total coal reserves and represent an important source of high-quality coking coal and anthracite^2^. However, the exploitation of these seams poses persistent and significant geotechnical challenges. Gravity-driven migration and asymmetric accumulation of fractured roof strata result in a highly non-uniform “upper void–lower solid” goaf structure. This structure induces uneven load distribution on powered supports and increases the risk of equipment instability^3^. As near-horizontal reserves decline, ensuring the stability of support structures in steeply dipping faces has become a critical requirement for safe and sustainable mining operations.

Research on support stability in inclined and steeply dipping coal seams has advanced significantly over the past two decades. Early studies primarily focused on determining the required support working resistance under static or quasi-static loading conditions. For gently inclined seams, reliable models have been developed for gob-side entry retention^4^, upward mining feasibility^5^, and overburden fracture evolution^6–8^. Concurrently, the “Roof–Support–Floor” (R–S–F) system dynamics framework provided fundamental insights into asymmetric load transfer mechanisms and failure patterns in steeply dipping seams^9–15^. These studies established empirical criteria for support capacity and identified the primary modes of strata movement. Further investigations have examined the structural characteristics of overlying strata in steeply dipping seams. Numerical simulations and theoretical analyses reveal that roof breakage produces inclined and counter-inclined stacking configurations, which together constitute a three-dimensional “shell-like” stope architecture that governs load transfer and support behavior^16,17^. Mechanical models developed for high mining height conditions have further established quantitative relationships between coal wall pressure and the support resistance required under spalling scenarios^18^. The influence of surface topography has also been incorporated into roof control frameworks through a combination of theoretical analysis, experimental simulation, and field measurement^19^. Moreover, composite roof models—exemplified by the “cantilever beam–masonry beam” structure—have been advanced to capture the coupled feedback between roof failure and coal wall spalling in high-production faces^20^.

More recent studies have shifted the focus from isolated support capacity to dynamic coupling between support equipment and the surrounding rock mass. Studies on coal–gangue interbedded roof instability and real-time monitoring of hydraulic support behavior indicate that sliding, toppling, and uneven loading are interconnected phenomena rather than independent failures^21,22^. Despite these advances, critical gaps remain in the literature. First, most mechanical models for support stability assume an idealized straight-line working face layout. This assumption fails to capture the geometric and mechanical advantages of curved (arc-section) face configurations, which are increasingly used to enhance stability. Second, although the adverse effects of dip angles exceeding 35° on support behavior are qualitatively recognized, quantitative comparisons of sliding and toppling stability between inclined straight sections and curved transition sections under identical conditions are lacking. Consequently, a unified analytical framework integrating curved face geometry with both anti-sliding and anti-toppling criteria has not yet been established.

Despite these advances, two key issues remain. First, most existing mechanical models assume a straight-line working face layout and fail to capture the advantages of curved (arc-section) configurations. Second, although the adverse effects of steep dip angles (> 35°) on support sliding and toppling are qualitatively recognized, quantitative comparisons between inclined straight and curved transition sections under identical conditions are lacking. Consequently, a unified analytical framework integrating curved face geometry with both anti-sliding and anti-toppling criteria has not yet been established.

The engineering necessity of this study is demonstrated by conditions observed prior to implementing a curved layout at Longwall Top Coal Caving (LTCC) Face 32,213, where the seam dip is 36°. Before optimization, four interrelated stability issues were identified: (1) support sliding during relocation due to increased gravitational components; (2) support toppling caused by overturning moments exceeding stabilizing moments; (3) downward sliding of the scraper conveyor due to thrust and self-weight; and (4) non-uniform support resistance distribution caused by asymmetric goaf filling. These issues are coupled, with sliding acting as the primary trigger for cascading instability affecting both toppling and conveyor alignment. This observation indicates that controlling support sliding is the key to effective stability management. Therefore, a systematic analysis incorporating curved geometry into stability calculations is required to address these issues.

To address these gaps, this study develops and validates a unified mechanical framework for assessing support stability in steeply inclined working faces. The main contributions of this study are threefold: (1) deriving critical stability equations for sliding and toppling in inclined straight and curved sections under optimal roof-contact conditions; (2) determining the minimum yield resistance required to maintain stable support configurations; and (3) demonstrating, through theoretical analysis and field validation, that curved face geometries improve stress distribution in the lower section by reducing the effective inclination angle.

## Engineering geological overview and adaptability of arc segment arrangement

**Fig. 1:**
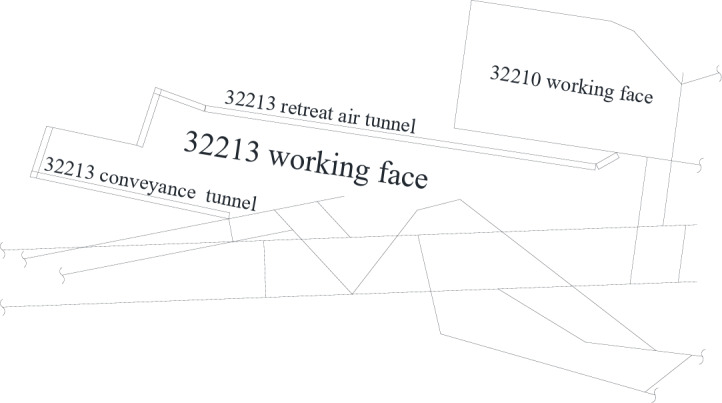
Layout of working face.

**Fig. 2:**
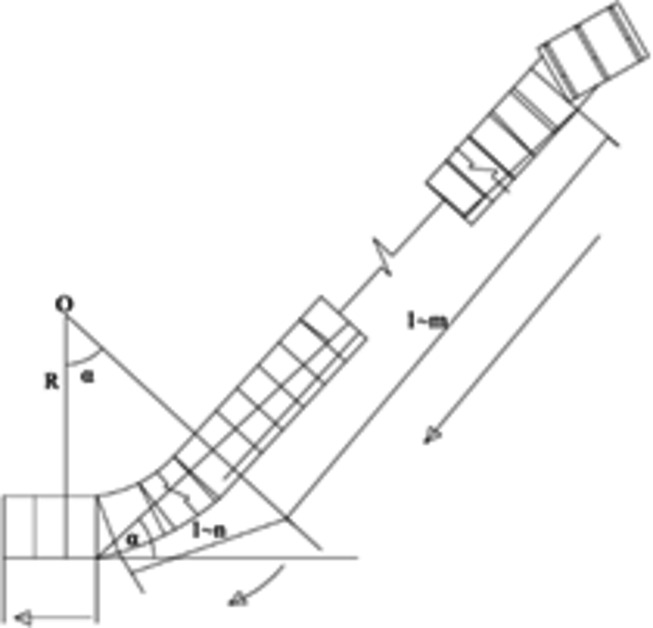
Schematic of powered support configuration in mining face.

The mining enterprise is situated in Pingliang City, Gansu Province. As shown in Figs. 1 and 2^3^, Longwall Face 32,213 is the first working face in the lower section of No. 3 Mining Area, with Coal 2# as the primary seam. The total thickness of Coal 2# in Face 32,213 is 10.20 m, characterized by a simple and stable coal structure. The immediate roof of Coal 2# is composed of 4.20 m of silty sandstone, overlaid by 10.19 m of medium-fine sandstone as the main roof. The immediate floor consists of 9.04 m of siltstone, with 4.71 m of fine sandstone forming the main floor.

The arc section layout connects the horizontal and inclined sections through a transition, reducing the inclination angle of the working face, enhancing the stability of the support system, and decreasing the failure rate of the scraper conveyor. It also simplifies the support layout at both the upper and lower ends, ensuring production safety and efficiency. However, the layout of the arc section is significantly influenced by factors such as the coal seam dip angle and thickness. Analyzing the occurrence conditions of the 32,213 working face is essential.

**Fig. 3:**
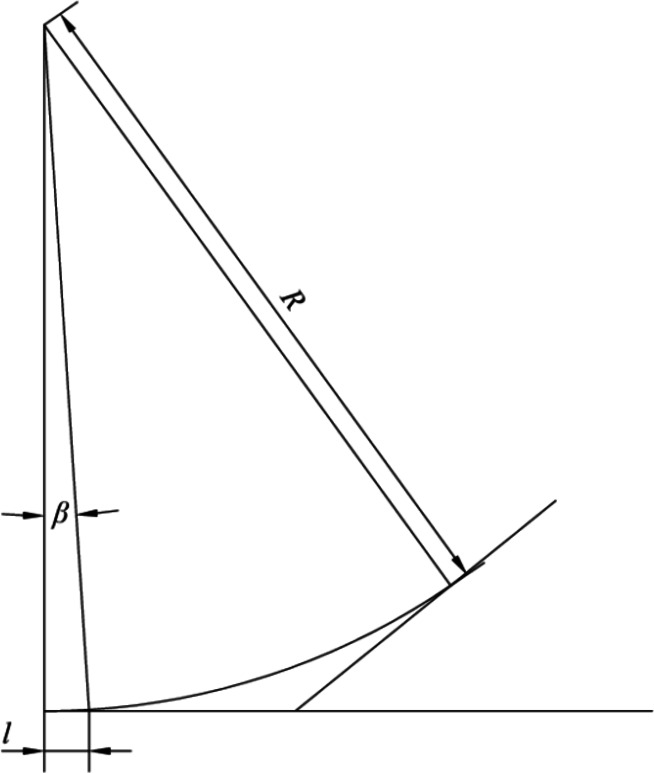
Relationship between arc radius and scraper conveyor parameters.

The minimum radius of the arc section depends on the vertical curvature of the scraper conveyor and the length of the chute, as shown in Fig. 3. From the geometric relationship, it can be obtained that:

1 $$R=\frac{l}{{2\sin \left( {\frac{\beta }{2}} \right)}}$$

In the formula,

*R-* the radius of the arc(m);

*l-* The length of one section of chute(m);

*β-* Vertical bending degree of the plate conveyor (°).

Among them, *β* = *α*/(*n* + 1), n is the number of chute sections. When *n*=(*α*/*β*)-1 is an integer and the vertical bending degree of the scraper conveyor takes the maximum value, the minimum arc radius that satisfies the maximum bending degree is (2).

2 $$\left[ R \right]=\frac{l}{{2\sin \frac{\alpha }{{2\left[ {INT\left( {\frac{\alpha }{{\left[ \beta \right]}} - 1} \right)+1} \right]}}}}$$

In the formula,

[*β*]*-* The maximum vertical bending degree of the scraper conveyor (°);

[*R*]- The minimum arc radius corresponding to the maximum vertical curvature of the scraper conveyor(m);

*α*- Coal seam inclination (°).

**Fig. 4:**
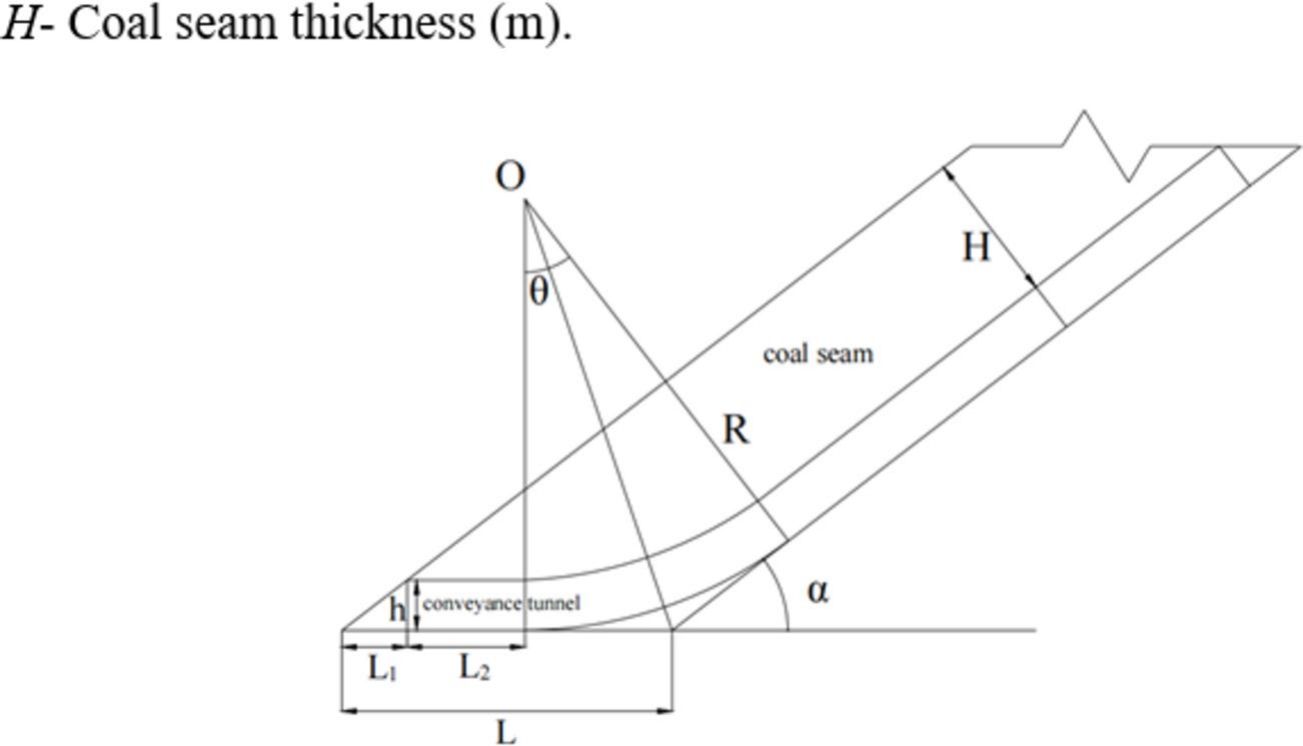
Layout of the lower arc section of the working face.

Establish the layout diagram of the lower arc section of the working face as shown in Fig. 4. According to the geometric relationship, it can be known that the relationship between the radius *R* of the arc section and the central angle *θ*, the inclination angle of the coal seam, the size of the machine roadway, and the thickness of the coal seam is:

3 $$R=\frac{{L - {L_1} - {L_2}}}{{\tan \frac{\theta }{2}}}$$

4 $$L=\frac{H}{{\sin \alpha }}$$

5 $${L_1}=\frac{h}{{\sin \alpha }}$$

In the formula,

*L*- Horizontal thickness of coal seam (m);

*L*_1_- The width of the triangular coal at the end of the machine tunnel (m);

*L*_2_- Width of the machine lane (m);

*h*- Height of the machine lane (m);

*H*- Coal seam thickness (m).

At this point, the radius of the arc segment obtained is the maximum radius. Only when the maximum radius *R*_max_ of the arc segment is greater than the minimum radius *R*_min_ of the arc segment can the arc transition condition be satisfied. The central angle is equal to the value of the coal seam dip angle. The coal seam dip angle *α* of the 32,213 working face is 36°, the coal seam thickness H is 10.2 m, and the machine roadway size is 4.0 m×2.5 m. Substituting the above parameters into Eq. (3) yields the maximum radius *R*_max_ of the arc segment is 30.51 m, which satisfies the arc transition condition.

The coal seam thickness conditions required for the circular arc section arrangement can be obtained by connecting (3) to (5) as follows:

6 $$H=\left( {{L_1}+{L_2}+\left[ R \right]\tan \frac{\theta }{2}} \right)\sin \alpha$$

Substituting the layout parameters of the coal seam arc section into the above formula, it is obtained that the coal seam thickness required to satisfy the minimum radius of the arc section should be 9.85 m. The minimum coal seam thickness *H*_min_ is less than the coal seam thickness *H*, which conforms to the layout conditions of the arc section.

Although the average seam thickness of 10.20 m satisfies the theoretical geometric requirement of 9.85 m for the curved transition layout, natural sedimentary processes introduce inherent variability in coal seam thickness. To ensure the robust applicability of the curved layout and to complete the geological suitability analysis, a set of proactive control measures and adaptive strategies is proposed for zones where the local thickness falls below 9.85 m.

Prior to face advance, a high-resolution seam thickness contour map will be generated using underground seismic tomography and in-seam drilling data to identify localized thinning zones. In sections where the seam thickness is slightly below 9.85 m but exceeds the minimum mining height of the equipment (e.g., 2.5 m for the maingate roadway), the following adaptive strategies will be implemented: (1) Controlled Cutting Adjustment: Minor floor dinting or roof brushing will be performed at the shearer level to maintain the required vertical curvature radius and clearance for the armored face conveyor, thereby ensuring the geometric integrity of the arc transition. (2) Variable-Height Support Utilization: Deploying two-leg shield supports with a wide adjustment range allows dynamic adaptation of support height, thereby accommodating local reductions in seam thickness without inducing instability due to poor roof contact. (3) Dynamic Curvature Optimization: If a more extensive thin zone is encountered, the central angle and radius of the curved section can be locally adjusted (steepened) to reduce the minimum seam thickness required by Eq. (6), thereby preserving the mechanical benefits of the arc layout while accommodating geological constraints. By integrating predictive detection methods with operational flexibility, the feasibility of the curved layout is not solely dependent on the average seam thickness but is robustly ensured against local geological variability.

## Mechanical analysis of sliding instability of hydraulic supports

**Fig. 5:**
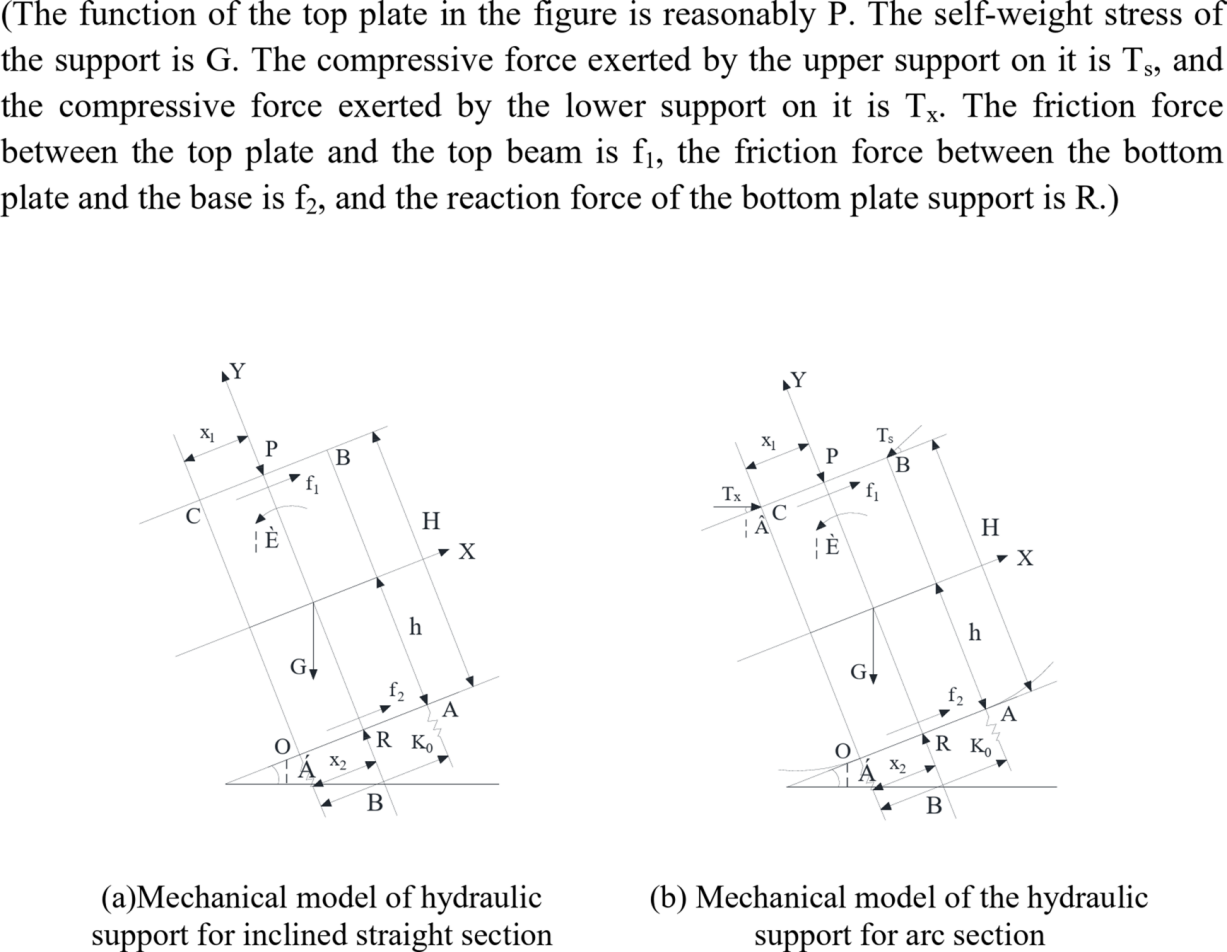
Mechanical model of hydraulic support for circular arc section of large inclined angle working face. (The function of the top plate in the figure is reasonably P. The self-weight stress of the support is G. The compressive force exerted by the upper support on it is T_s_, and the compressive force exerted by the lower support on it is T_x_. The friction force between the top plate and the top beam is f_1_, the friction force between the bottom plate and the base is f_2_, and the reaction force of the bottom plate support is R.)

The stability of both the inclined straight section and arc section supports in the “inclined straight - arc - horizontal” arrangement of the 32,213 working face is analyzed. Let the number of inclined straight section supports be m and the number of arc section supports be n. A mechanical model of the hydraulic support under normal conditions is developed, as shown in Fig. 5. A mechanical analysis of both the inclined section and arc section supports is conducted.

The support remains stable under the combined action of the resultant force *P* exerted by the top plate, the self-weight *G* of the support, the squeezing force T_S_ exerted by the upper adjacent support, the squeezing force T_X_ exerted by the lower adjacent support, the frictional force *f*_1_ between the top plate and the top beam, the frictional force *f*_2_ between the bottom plate and the base, and the reaction force *R* of the bottom plate support. Mechanical analysis was conducted on any support on the inclined straight section of the working face and the *i*-th support on the arc section starting from the top to obtain the conditions under which the support tends to maintain sliding stability along the working face.

Support for inclined straight section:

7 $$\left\{ \begin{gathered} {T_x}+{f_1}+{f_2} \geqslant G\sin \alpha +{T_s} \hfill \\ P+G\cos \alpha =R \hfill \\ \end{gathered} \right.$$

In the formula, *α* - the inclination angle of the coal seam(°).

Support for arc section:

8 $$\left\{ \begin{gathered} {T_x}\cos \beta +{f_1}+{f_2} \geqslant G\sin \left( {\alpha - i\beta } \right)+{T_s}\cos \beta \hfill \\ P+G\cos \left( {\alpha - i\beta } \right)+\left( {{T_x}+{T_s}} \right)\sin \beta =R \hfill \\ \end{gathered} \right.$$

In the formula, *β* - the angle between the supports of the circular arc segment(°).

When the support is in a critical state of instability, the frictional forces *f*_1_ and *f*_2_ between the support and the top and bottom plates reach their maximum. The frictional forces between the inclined straight section support and the circular arc section support and the top and bottom plates of the working face are as follows:

9 $$\left\{ {\begin{array}{*{20}{l}} {{f_{1inclined}}={\mu _1}P} \\ {{f_{2inclined}}={\mu _2}(P+G\cos \alpha )} \end{array}} \right.$$

10 $$\left\{ {\begin{array}{*{20}{l}} {{f_{1circle}}={\mu _1}P} \\ {{f_{2circle}}={\mu _2}(P+G\cos (\alpha - i\beta )+({T_x}+{T_s})\sin \beta )} \end{array}} \right.$$

In the formula,

*f*_1 inclined_ - Friction between the inclined straight section support and the top plate(kN);

*f*_2 inclined_ - Friction between the inclined straight section support and the base plate(kN);

*f*_1 circle_ - Friction between the arc section support and the top plate(kN);

*f*_2 circle_ - Friction between the arc section support and the top plate(kN);

*µ*_1_ - The friction coefficient between the top plate and the support(kN);

*µ*_2_ - The friction coefficient between the base plate and the support(kN).

From Eq. (7) to (10), the critical working resistances for maintaining stability of the inclined straight section support and the arc section support on the working face can be obtained as follows:

11 $$\left\{ {\begin{array}{*{20}{l}} {{P_{hinclined}}=\frac{{G\sin \alpha +{T_s} - {T_x} - {\mu _2}G\cos \alpha }}{{{\mu _1}+{\mu _2}}}} \\ {{f_{hcircle}}=\frac{{G\sin (\alpha - i\beta ) - ({T_x} - {T_s})\cos \beta - {\mu _2}(G\cos (\alpha - i\beta )+({T_x}+{T_s})\sin \beta )}}{{{\mu _1}+{\mu _2}}}} \end{array}} \right.$$

In the formula,

*P*_h inclined_- The critical working resistance of the inclined straight section support when it is in a critical sliding state(kN);

*P*_h circle_ - The critical working resistance of the arc-shaped support when it is in a critical sliding state(kN).

### Analysis of anti-slip stability of a single support

When the support is moved, the constraint effect of the top plate on it will weaken or disappear, and the support will be in an empty top state. When the upper part of the top beam loses its constraint, *P* = 0, and the squeezing force *T*_x_*=T*_s_*=*0 exerted by adjacent supports on it. The supports remain balanced under their own conditions. Substituting into Eqs. (7) and (8), the conditions for the inclined straight section supports and the arc section supports to maintain sliding stability can be obtained.

Support for inclined straight section:

12 $$\left\{ \begin{gathered} {\mu _2}G\cos \alpha \geqslant G\sin \alpha \hfill \\ G\cos \alpha =R \hfill \\ \end{gathered} \right.$$

Support for arc section:

13 $$\left\{ \begin{gathered} {\mu _2}G\cos \left( {\alpha - i\beta } \right) \geqslant G\sin \left( {\alpha - i\beta } \right) \hfill \\ G\cos \left( {\alpha - i\beta } \right)=R \hfill \\ \end{gathered} \right.$$

After sorting, the critical sliding angles of the inclined straight section support and the arc section support are as follows:

14 $$\left\{ \begin{gathered} {\alpha _1}=\arctan \left( {{\mu _2}} \right) \hfill \\ {\alpha _2}=\arctan \left( {{\mu _2}} \right)+i\beta \hfill \\ \end{gathered} \right.$$

In the formula,

*α*_1_- The critical sliding angle of a single support in the inclined straight section (°);

*α*_2_- The critical sliding angle of a single support in the arc section (°).

The stable critical sliding angle of a single support in the overhead state is influenced by the friction coefficient between the support and the base plate, as well as the slope start angle. Focusing on the topmost support of the arc section, the friction coefficient between the support and the base plate was substituted into the critical instability angle formula, deriving the relationship between the critical sliding angle and the friction coefficient.

It can be inferred that the critical sliding angle of the support in the overhead state increases with the friction coefficient between the support and the base plate. Since the critical sliding angle of the support is much smaller than the inclination angle of the working surface, an anti-slip force must be applied to the support.

When the support is fully connected (*P* ≠ 0), it tends to slide downwards due to the downward force along the working surface. The support remains stable when the anti-sliding force is greater than or equal to the sliding force. At this point, the critical working resistance for both the inclined straight section and the arc section supports is:

15 $$\left\{ \begin{gathered} {P_{hinclined}}=\frac{{G\sin \alpha - {\mu _2}G\cos \alpha }}{{{\mu _1}+{\mu _2}}} \hfill \\ {P_{hcircle}}=\frac{{G\sin \left( {\alpha - i\beta } \right) - {\mu _2}G\cos \left( {\alpha - i\beta } \right)}}{{{\mu _1}+{\mu _2}}} \hfill \\ \end{gathered} \right.$$

**Fig. 6:**
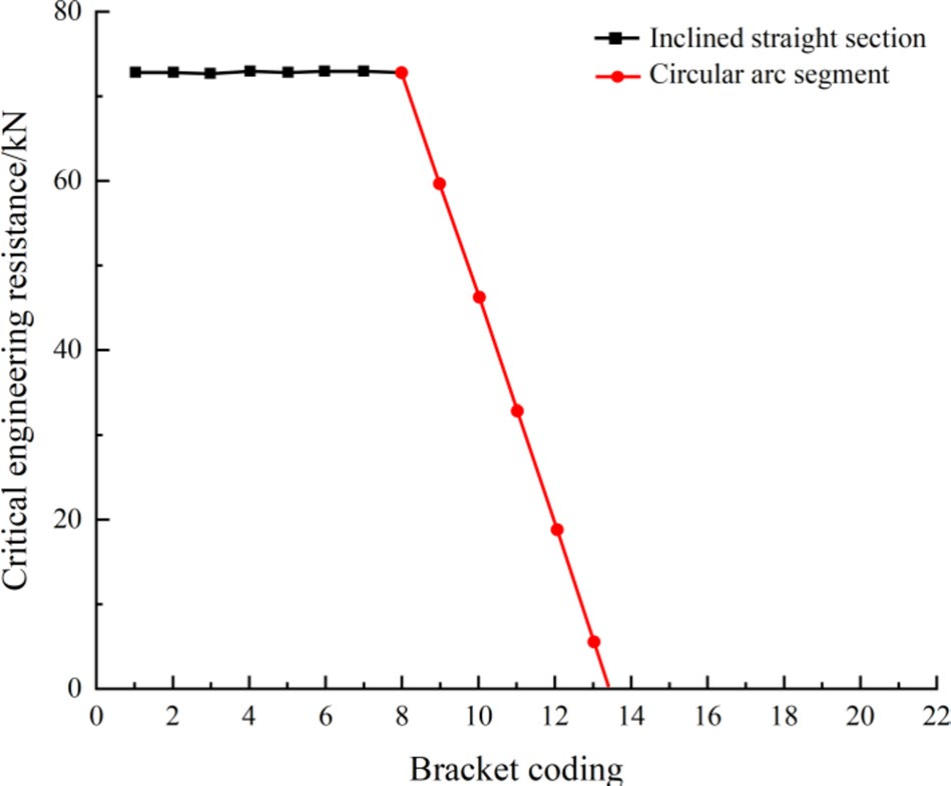
Graph of the critical anti-slip working resistance variation of the support along the working surface.

The friction coefficient *µ*_1_ between the top plate and the support is 0.35, the friction coefficient *µ*_2_ between the bottom plate and the support is 0.36 (The coefficients were obtained through extensive laboratory direct shear tests on coal and rock samples collected by Face 32213, as well as field-specific experimental measurements of frictional resistance between typical hydraulic support substrates and adjacent siltstone floors.), the self weight *G* of the support is 175kN, and the starting angle *β* is 3°. Substitute the above parameters into Eq. (15) and draw the variation of the critical working resistance of the support along the inclination of the working face, as shown in Fig. 6.

From the analysis of Fig. 6, it can be seen that as the inclination angle of the working face increases, the critical working resistance of the support also increases. When the support is in the no-top state, the critical sliding instability angle is approximately 20°. When the support is in the fully connected top state, the critical sliding working resistance of the inclined straight section support remains constant, while that of the arc section support decreases linearly. The critical sliding working resistance of the support on the working face is approximately 73 kN.

Let the stability coefficient of the support be *K*, and the anti-slip coefficient *K*_*h*_ be the ratio of the anti-slip resultant force to the sliding resultant force.

1) Anti-slip coefficient of the inclined straight section support in the critical state:

16 $$\left\{ \begin{gathered} {K_{h1}}={\mu _2}\cot \alpha \hfill \\ {K_{h2}}={K_{h1}}+\frac{{P\left( {{\mu _1}+{\mu _2}} \right)}}{{G\sin \alpha }} \hfill \\ \end{gathered} \right.$$

2) Anti-slip coefficient of the arc section support in the critical state:

17 $$\left\{ \begin{gathered} {K_{h1}}={\mu _2}\cot \left( {\alpha - i\beta } \right) \hfill \\ {K_{h2}}={K_{h1}}+\frac{{P\left( {{\mu _1}+{\mu _2}} \right)}}{{G\sin \left( {\alpha - i\beta } \right)}} \hfill \\ \end{gathered} \right.$$

In the formula,

*K*_h1_- The anti-slip coefficient of the support under the no-roof state;

*K*_h2_- The anti-slip coefficient of the support under the state of full top connection.

From Eqs. (16) and (17), it can be seen that the anti-sliding stability coefficient of a single support is influenced by the roof pressure on the support, the self-weight of the support, the inclination angle of the coal seam, and the friction coefficient between the roof, floor, and the support.

**Fig. 7:**
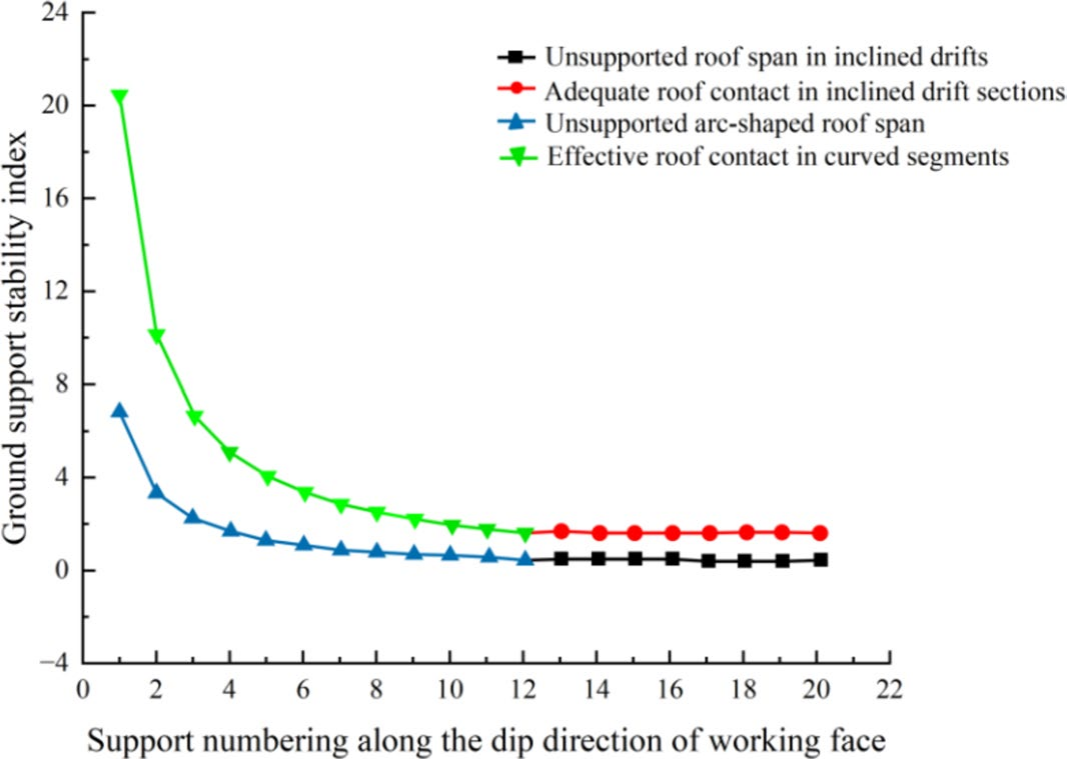
Variation of anti-slip stability coefficient of support inclination along the working face.

When the resultant force acting on the top plate of the support is constant, the variation of the anti-slip stability coefficient along the working surface, from top to bottom, is shown in Fig. 7.

Figure 7 shows that the anti-slip stability coefficients of both the inclined straight section support and the arc section support are lower in the empty top state than in the fully connected top state. As the number of supports increases, the anti-slip stability coefficient of the arc section support decreases continuously, following an inverted parabolic shape, while that of the inclined straight section support remains constant. This indicates that the anti-slip stability of the lower support of the arc section is relatively high, while the upper support still carries a significant risk of sliding instability.

The “empty roof section” in the asymmetric accumulation structure formed by mining steeply inclined coal seams is located at the upper part of the working face (the inclined straight section). Therefore, only the case of an empty roof at the upper end support of the inclined straight section is considered. Let *j* represent the number of empty roof supports above a certain support in the inclined straight section. Assuming the support below exerts no thrust on it, the critical anti-slip working resistance of this support can be expressed as:

18 $$P=\frac{{\left( {j+1} \right)\left( {G\sin \alpha - {\mu _2}G\cos \alpha } \right)}}{{{\mu _1}+{\mu _2}}}$$

**Fig. 8:**
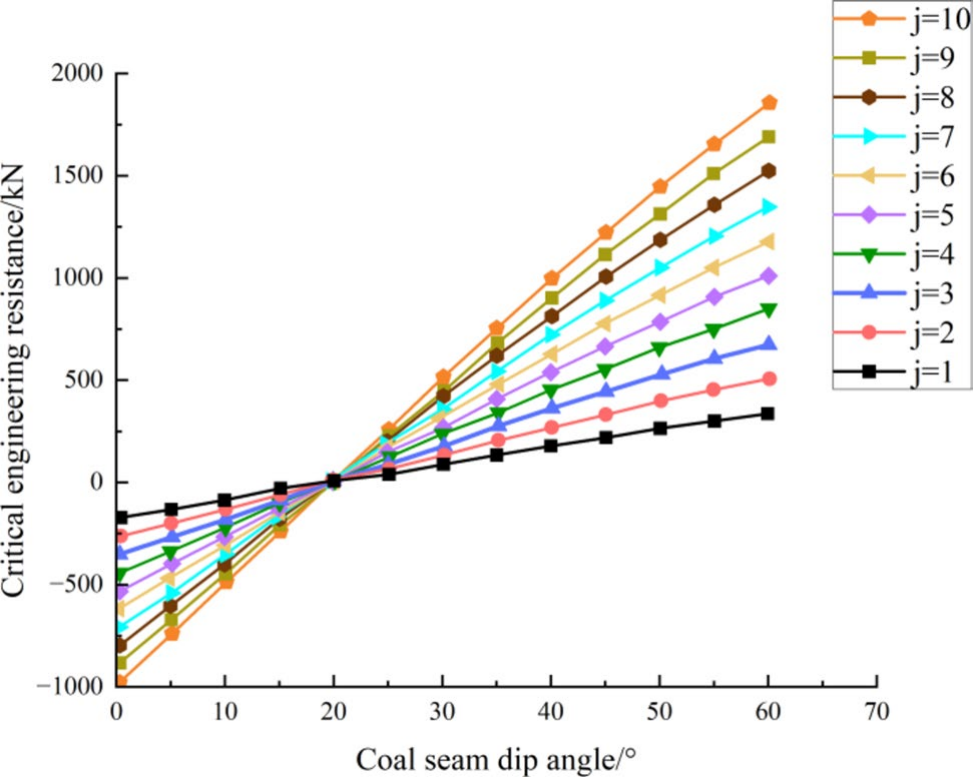
Influence of coal seam inclination on critical anti-slip.

Let the number of overhead supports *j* range from 1 to 10. The relationship between the critical working resistance of the supports and the inclination angle of the coal seam is illustrated in Fig. 8. From the analysis of Fig. 8, it can be inferred that when the coal seam inclination is approximately 19.8°, the support can maintain sliding stability on its own. As the coal seam inclination angle increases, the critical anti-sliding working resistance of the support increases linearly. When the coal seam inclination angle is constant, the greater the number of open-top supports above the support, the greater the anti-sliding working resistance required to maintain stability.

working resistance of braces.

### Analysis of the anti-slip stability of the overall support system

During the mining process, the supports are not only closely related to the top and bottom plates, but also the interaction between the supports has a significant impact on the stability of the supports. When a certain stent becomes unstable, the squeezing force *T*_*s*_ exerted by the adjacent stent above it is 0. When the stent slides down, it will exert a squeezing force on the adjacent stent below. For the same stent, *T*_s_≠*T*_x_. Since the squeezing forces between adjacent stents are equal, that is, *T*_si_=*T*_*x*(*i−*1)_, (*i* = 1 ~ *n*).

(1) Taking the first support at the upper end of the inclined straight section as the research object, considering the bottom plate as a rigid body, and taking the critical state of the support as the condition for the sliding instability of the support, when the top connection is sufficient, the first support slides down, and the lateral force it exerted on the second support is:

19 $${T_{hs2}}=G\sin \alpha - {\mu _1}P - {\mu _2}\left( {G\cos \alpha +P} \right)$$

The lateral force acting on the third support:

20 $${T_{hs3}}=2\left[ {G\sin \alpha - {\mu _1}P - {\mu _2}\left( {G\cos \alpha +P} \right)} \right]$$

By the same token, the lateral force acting on the (i + 1)- th support is:

21 $${T_{hs\left( {i+1} \right)}}=i\left[ {G\sin \alpha - {\mu _1}P - {\mu _2}\left( {G\cos \alpha +P} \right)} \right]$$

(2) Taking the first support at the upper end of the arc section as the research object, considering the bottom plate as a rigid body, and taking the critical state of the support as the condition for the instability of the support, when the top connection is sufficient, the first support slides down, and the lateral force it exerted on the second support is:

22 $${T_{hs2}}=\frac{G}{{\cos \beta }}\sin \left( {\alpha - \beta } \right) - \frac{{\left( {{\mu _1}+{\mu _2}} \right)P}}{{\cos \beta }} - \frac{{{\mu _2}G}}{{\cos \beta }}\cos \left( {\alpha - \beta } \right) - {\mu _2}{T_{x1}}\tan \beta$$

The lateral force exerted by the second support on the third support is:

23 $$\begin{gathered} {T_{hs3}}=\frac{G}{{\cos \beta }}\left[ {\sin \left( {\alpha - \beta } \right)+\sin \left( {\alpha - 2\beta } \right)} \right] - \\ \frac{{{\mu _2}G}}{{\cos \beta }}\left[ {\cos \left( {\alpha - \beta } \right)+\cos \left( {\alpha - 2\beta } \right)} \right] - \\ {\mu _2}\left( {{T_{x1}}+{T_{x2}}+{T_{s2}}} \right)\tan \beta - \frac{{2\left( {{\mu _1}+{\mu _2}} \right)P}}{{\cos \beta }} \\ \end{gathered}$$

Similarly, the lateral force exerted by the i-th support on the (i + 1) th support is:

24 $$\begin{gathered} {T_{hs\left( {i+1} \right)}}=\frac{G}{{\cos \beta }}\left[ {\sin \left( {\alpha - \beta } \right)+\sin \left( {\alpha - 2\beta } \right)+ \cdot \cdot \cdot \cdot \cdot \cdot +\sin \left( {\alpha - i\beta } \right)} \right] - \\ \frac{{{\mu _2}G}}{{\cos \beta }}\left[ {\cos \left( {\alpha - \beta } \right)+\cos \left( {\alpha - 2\beta } \right)+ \cdot \cdot \cdot \cdot \cdot \cdot +\cos \left( {\alpha - i\beta } \right)} \right] - \\ \frac{{i\left( {{\mu _1}+{\mu _2}} \right)P}}{{\cos \beta }} - {\mu _2}\left( {\sum\limits_{{n=1}}^{i} {{T_{xn}}+\sum\limits_{{n=2}}^{i} {{T_{sn}}} } } \right)\tan \beta \\ \end{gathered}$$

The compressive force exerted by the upper side support on the first support on the arc section is the compressive force exerted by the first support on the arc section on the last support of the inclined straight section. From this, the mechanical relationship of the pressure exerted by the roof on the *i*-th support on the arc section can be obtained as:

25 $$\begin{gathered} G\left[ {\sin \left( {\alpha - \beta } \right)+\sin \left( {\alpha - 2\beta } \right)+ \cdot \cdot \cdot +\sin \left( {\alpha - i\beta } \right)} \right]+{T_{s1}}\cos \beta = \hfill \\ {\mu _2}\sin \beta \left( {{T_{x1}}+2{T_{x2}}+ \cdot \cdot \cdot +2{T_{x\left( {i - 1} \right)}}+{T_{xi}}} \right)+i{\mu _1}P+i{\mu _2}P+ \hfill \\ {T_{xi}}\cos \beta +{\mu _2}G\left[ {\cos \left( {\alpha - \beta } \right)+\cos \left( {\alpha - 2\beta } \right)+ \cdot \cdot \cdot +\cos \left( {\alpha - i\beta } \right)} \right] \hfill \\ \end{gathered}$$

Specifically, as derived in Eq. (24), the lateral force exerted by the *i*-th support on its lower neighbor represents the sum of unbalanced down-dip force components from all overlying supports. n the curved section, the cumulative lateral thrust *T*_*x*(*i*)_ as an external disturbance on the *i*-th support, tending to induce rotational or translational displacement of the support canopy relative to the roof. To maintain static equilibrium and ensure full roof contact (a prerequisite for the model boundary conditions), the support must generate a normal reaction force *P*_*i*_. This required force is determined not only by the local overburden weight but also by the need to counteract the destabilizing moment and sliding potential induced by *T*_*x*(*i*)_. Consequently, the roof pressure *P*_*i*_ in Eq. (25) is obtained by solving the coupled force and moment equilibrium equations for the *i*-th support under the combined effects of gravity, roof pressure, floor reaction, and the cumulative lateral constraint *T*_*x*(*i*)_ transmitted from the overlying supports.

Among them:

26 $${T_{s1}}=mG\sin \alpha - m{\mu _1}P - m{\mu _2}\left( {G\cos \alpha +P} \right)$$

**Fig. 9:**
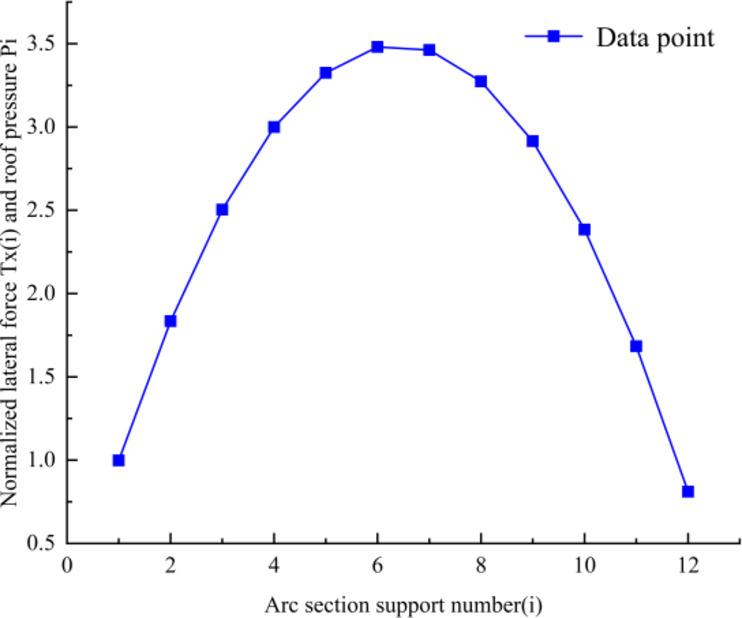
Trend chart of lateral force and roof pressure changes.

From Equations (19) to (26), it can be seen that the lateral extrusion force on a support in both the inclined straight section and arc section is the sum of the lateral extrusion force from the upper support. The inclination angle of the inclined straight section support remains constant, and both the lateral extrusion force and the roof pressure increase linearly. The inclination angle of the arc section support gradually decreases from top to bottom. Based on the trigonometric function relationship within the inclination angle range, it can be inferred that the lateral force and roof pressure on the arc section support decrease in a parabolic shape(Fig. 9). The arrangement of the arc sections improves the stress distribution of the lower support and reduces the risk of continuous sliding instability.

### Quantitative analysis of the influence of arc section optimization on anti-slip stability

Based on the anti-slip stability coefficients *K*_*h*_ defined in Eqs. (16) and (17), the anti-slip coefficients of the supports at different positions in the inclined straight and arc sections are calculated under the same roof pressure conditions. The calculation results show that:

The anti-slip coefficient *K*_*h*_, _*straight*_ of the inclined straight section support (with an inclination Angle of 36°). *K*_*h*_, _*straight*_=0.76, is in a critical unstable state;

Upper end support of the arc-shaped section (initial inclination Angle 36°, initial Angle β = 3°) The anti-slip coefficient is *K*_*h, arc−upper*_=0.82, an increase of 7.9% compared to the straight line segment;

The anti-slip coefficient of the middle support in the arc-shaped section (with an inclination Angle of approximately 18°) is *K*_*h, arc−middle*_=1.21, It has increased by 59.2% compared to the straight line segment.

The anti-slip coefficient of the lower end support of the arc-shaped section (with an inclination Angle approaching 0°) is *K*_*h, arc*−lower_=1.68, which is 121% higher than the straight line segment.

It can be seen that the arc-shaped arrangement, by gradually reducing the working inclination Angle of the support, causes the anti-slip stability coefficient to increase nonlinearly along the working surface, effectively suppressing the downward trend of the support. The theoretical calculations align with on-site observations: no significant sliding displacement was observed during the movement of the arc section support, while the average sliding of the inclined straight section support is approximately 35 mm per cycle.

## Mechanical analysis of Instability caused by the tilting of hydraulic supports

Mechanical analysis was conducted on any support on the inclined straight section of the working face and the *i*-th support on the arc section starting from the top to obtain the conditions for the support to maintain stable tilting along the tendency of the working face.

Support for inclined straight section:

27 $${f_1}H+{T_x}H+G\cos \alpha \frac{B}{2}+P{x_1} \geqslant G\sin \alpha h+{T_s}H+R{x_2}$$

Support for arc section:

28 $$\begin{gathered} {f_1}H+{T_x}\cos \beta H+G\cos \left( {\alpha - i\beta } \right)\frac{B}{2}+P{x_1}+\left( {{T_x}+{T_s}} \right)\sin \beta \frac{B}{2} \geqslant \hfill \\ G\sin \left( {\alpha - i\beta } \right)h+{T_s}\cos \beta H+R{x_2} \hfill \\ \end{gathered}$$

In the formula,

*H*- the height of the support (m);

*x*_1_- The lever arm between the top plate force and point *O* at the lower end of the support(m);

*B*- Support width (m);

*x*_2_- The lever arm between the reaction force of the base plate support and point *O* at the lower end of the support(m);

*h*- The height of the center of gravity of the support(m).

When the support is in the critical state of instability, the frictional forces *f*_1_ and *f*_2_ between the support and the top and bottom plates reach the maximum. From Eqs. (27) and (28), the critical working resistances for the support in the inclined straight section and the arc section of the working surface to maintain stability can be obtained as follows:

29 $$\left\{ \begin{gathered} {P_q}_{{inclined}}=\frac{{2G\sin \alpha h+\left( {{T_s} - {T_x}} \right)H+2R{x_2} - G\cos \alpha B}}{{2\left( {{x_1}+{\mu _1}H} \right)}} \hfill \\ {P_{qcircle}}=\frac{{2G\sin \left( {\alpha - i\beta } \right)h - G\cos \left( {\alpha - i\beta } \right)B - 2\left( {{T_x} - {T_s}} \right)\cos \beta H - \left( {{T_x}+{T_s}} \right)\sin \beta B+2R{x_2}}}{{2\left( {{x_1}+{\mu _1}H} \right)}} \hfill \\ \end{gathered} \right.$$

In the formula,

*P*_*q*_ inclined- The critical working resistance of the inclined straight section support when it is in a critical tilting state(kN);

*P*_*q*_ circle- The critical working resistance when the arc section support is in a critical tilting state(kN).

### Analysis of anti-tilt stability of a single scaffold

When the support is in the empty top state (*P* = 0), the squeezing forces *T*_x_=*T*_s_=0 exerted by adjacent supports on it, and the support remains balanced under its own conditions. Substituting into Eqs. (27) and (28), the conditions for the inclined straight section support and the circular arc section support to maintain anti-tilt stability can be obtained.

Support for inclined straight section:

30 $$\left\{ \begin{gathered} {\mu _2}G\cos \alpha \geqslant G\sin \alpha \hfill \\ G\cos \alpha =R \hfill \\ \end{gathered} \right.$$

Support for arc section:

31 $$\left\{ \begin{gathered} {\mu _2}G\cos \left( {\alpha - i\beta } \right) \geqslant G\sin \left( {\alpha - i\beta } \right) \hfill \\ G\cos \left( {\alpha - i\beta } \right)=R \hfill \\ \end{gathered} \right.$$

After sorting, the critical tipping angles of the inclined and straight section supports and the arc section supports are respectively:

32 $$\left\{ \begin{gathered} {\phi _1}=\arctan \left( {\frac{B}{{2h}}} \right) \hfill \\ {\phi _2}=\arctan \left( {\frac{B}{{2h}}} \right)+i\beta \hfill \\ \end{gathered} \right.$$

In the formula,

*Φ*_1_- The critical tipping angle of a single support in the inclined straight section(°);

*φ*_2_- The critical tipping angle of a single support in the arc section(°).

**Fig. 10:**
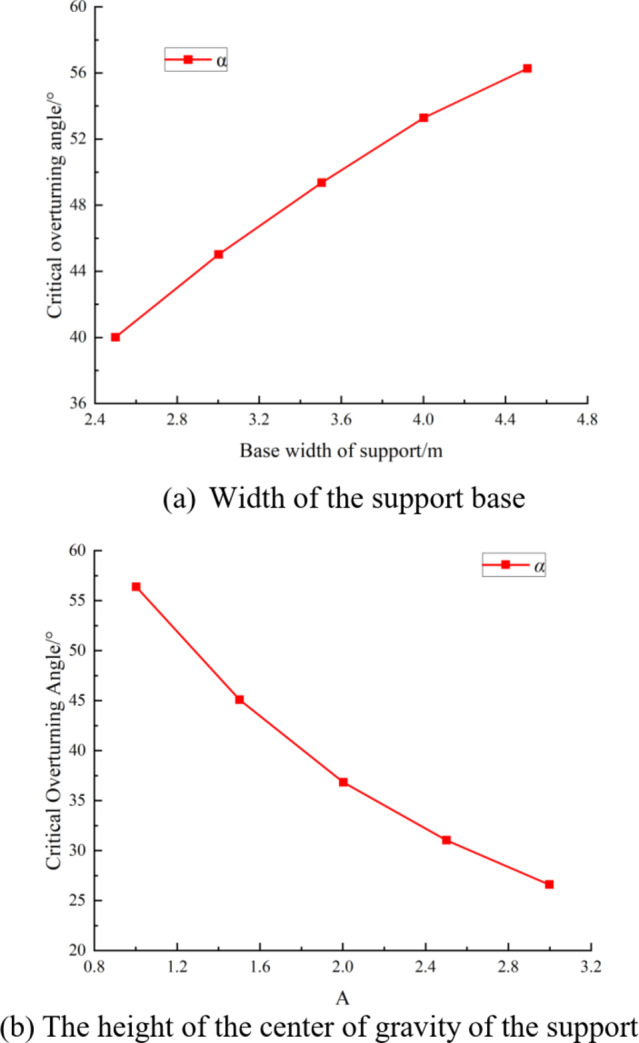
Influencing factors of the critical tipping angle of a single support.

The critical tipping angle at which a single support maintains stability in the overhead state is related to the slope start angle, the width of the support, and the height of the center of gravity of the support. Substituting the width of the support and the height of the center of gravity of the support into the critical tilt angle relationship formula of the support, the relationship between the critical tilt angle of the support and the width of the support and the height of the center of gravity of the support is obtained, as shown in Fig. 10. It can be known from Fig. 10 that the critical tilting angle of the support in the empty top state increases with the increase of the width of the support base and decreases with the increase of the center of gravity height of the support. Since the critical tipping angle of the support is much smaller than the inclination angle of the working surface, it is necessary to provide an anti-tipping force for the support. Meanwhile, in actual production, the width of the base of the support can be appropriately increased and the height of the center of gravity of the support can be reduced to improve the stability of the support.

When the support is in a fully jacking state (*P* ≠ 0), the support has a tendency to tip under the action of the downward component force along the working face. When the anti-tipping moment is greater than or equal to the tipping moment, the support remains stable. When the support is in the critical state of toppling, the reaction force of the bottom plate support acts on point *O* at the lower left end of the support, and the pressure *P* of the top plate acts on the upper right end of the support. The critical working resistance of the support is:

33 $$\left\{ \begin{gathered} {P_q}_{{inclined}}=\frac{{2G\sin \alpha h - G\cos \alpha B}}{{2\left( {{\mu _1}H+B} \right)}} \hfill \\ {P_{qcircle}}=\frac{{2G\sin \left( {\alpha - i\beta } \right)h - G\cos \left( {\alpha - i\beta } \right)B}}{{2\left( {{\mu _1}H+B} \right)}} \hfill \\ \end{gathered} \right.$$

**Fig. 11:**
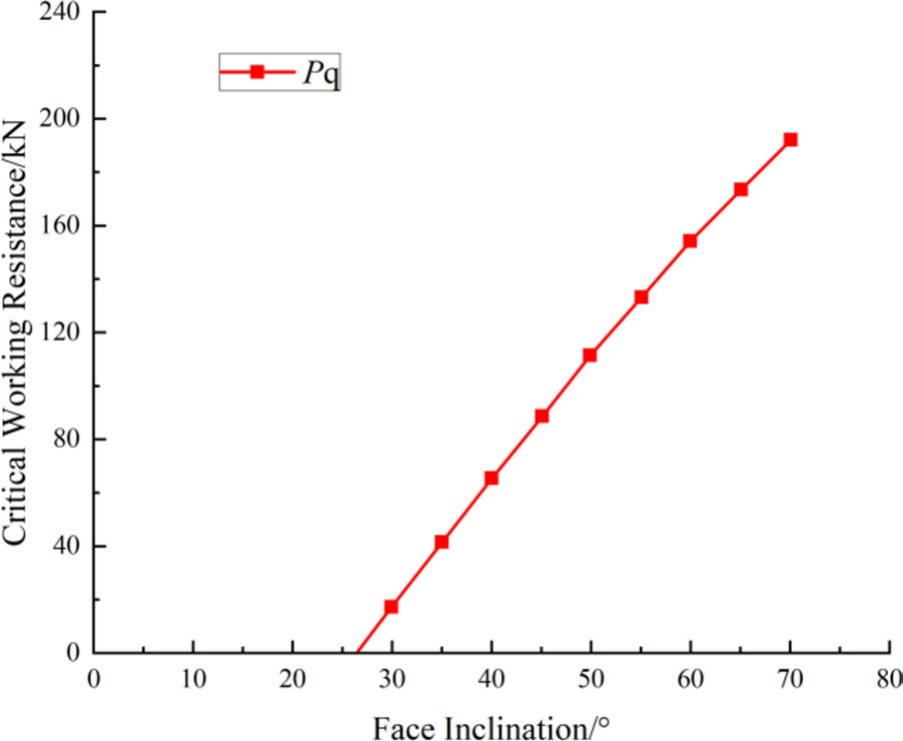
Relationship between critical working resistance of brace tipping and working face inclination angle.

**Fig. 12:**
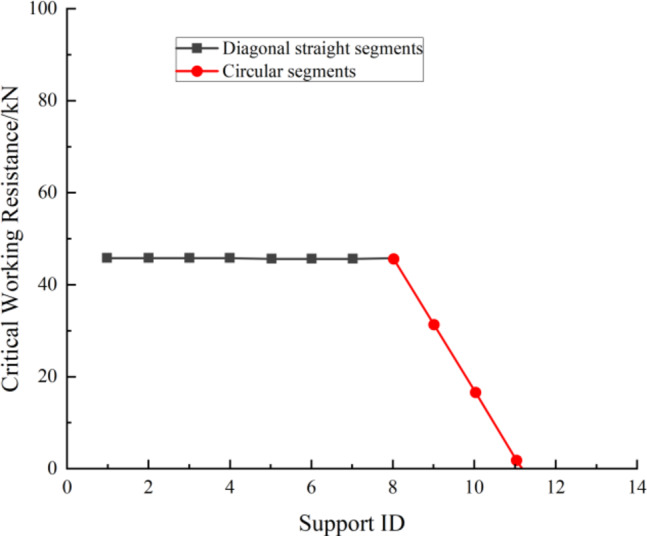
Variation of critical anti-tilting work resistance of the support along the working face inclination.

The height of the support frame *H* is 3 m, the center of gravity height of the support frame *h* = *H*/2 = 1.5 m, the slope start angle *β* is 3°, the friction coefficient *µ*_1_ between the top plate and the support frame is 0.35, the width of the support frame *B* is 1.5 m, and the self-weight of the support frame is 175kN. Substitute the above parameters into Eq. (33), and draw the relationship graph between the critical working resistance of the support tilting and the inclination angle of the working face, as well as the variation graph of the critical working resistance of the support tilting along the working face, as shown in Figs. 11 and 12.

Figures 11 and 12 show that as the inclination angle of the working face increases, the critical tilting resistance of the support correspondingly increases. When the support is in the no-top state, the critical angle for the support to tip over and become unstable is approximately 26.5°. When the support is in the fully connected top state, the critical tilting working resistance of the inclined and straight section support remains unchanged, while the critical tilting working resistance of the arc section support shows a linear decreasing trend. The critical sliding working resistance of the support on the working face is approximately 45.8kN. When the inclination angle of the working face is constant, the critical sliding working resistance of the support is greater than the critical tilting working resistance.

Let the stability coefficient of the support be *K*, and the anti-tilting coefficient *K*_q_ be the ratio of the anti-tilting moment to the tilting moment.

1. Anti-tilting coefficient of the inclined straight section support in the critical state:

34 $$\left\{ \begin{gathered} {K_{q1}}=\frac{B}{{2h}}\cot \alpha \hfill \\ {K_{q2}}={K_{q1}}+\frac{{P\left( {{\mu _1}H+B} \right)}}{{G\sin \alpha h}} \hfill \\ \end{gathered} \right.$$

(2) Anti-tilting coefficient of the arc section support in the critical state:

35 $$\left\{ \begin{gathered} {K_{q1}}=\frac{B}{{2h}}\cot \left( {\alpha - i\beta } \right) \hfill \\ {K_{q2}}={K_{q1}}+\frac{{P\left( {{\mu _1}H+B} \right)}}{{G\sin \left( {\alpha - i\beta } \right)h}} \hfill \\ \end{gathered} \right.$$

In the formula, *K*_q1_- The anti-tilting coefficient of the support under the no-roof state; *K*_q2_- Anti-tilting coefficient of the support under the fully connected top state.

It can be known from Eqs. (34) and (35) that the anti-tilt stability coefficient is related to the pressure of the roof on the support, the self-weight of the support, the inclination angle of the coal seam, the friction coefficient between the roof and floor and the support, the width of the support, and the height of the center of gravity of the support. Since the self-weight of the support and the roof pressure cannot be negative, the anti-tilting stability coefficient of the support in the empty roof state is smaller than that in the fully connected roof state. Appropriately increasing the working resistance of the support is conducive to maintaining its stability.

**Fig. 13:**
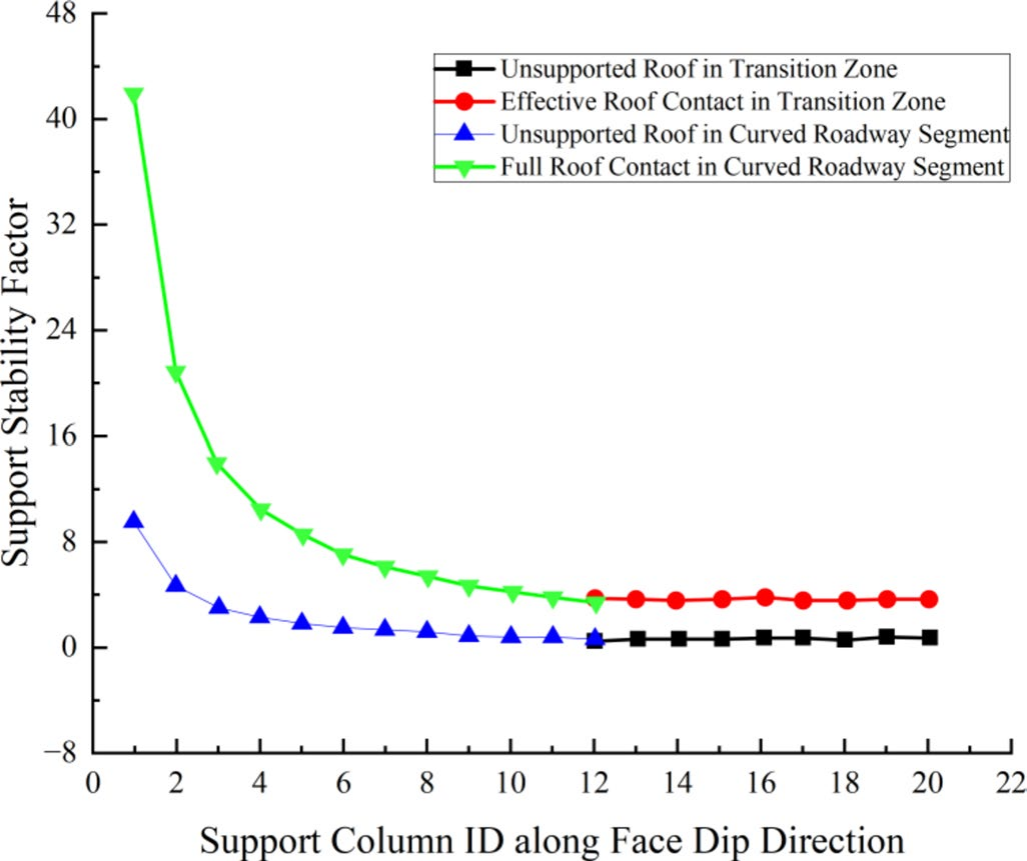
Variation of tilt stability coefficient of support. inclination along the working face.

When the resultant force acting on the top plate on the support is constant, the variation of the anti-tilt stability coefficient of the support from top to bottom along the working face is shown in Fig. 13.

It can be seen from Fig. 13 that the anti-tilting stability coefficients of the inclined straight section support and the arc section support in the empty top state are both less than those in the fully connected top state. With the increase of the support number, the anti-tilting stability coefficient of the arc section support continuously decreases, presenting an inverted parabolic shape, while the anti-tilting stability coefficient of the inclined straight section support remains unchanged. It can be known from this that the lower support along the inclined arc section has a relatively high anti-tilting stability, while the upper support along the arc section still has a relatively large risk of tilting instability. It can be known from Fig. 4 that in the “slant straight - arc - horizontal” arrangement of the large-inclination coal seam working face, under the empty roof state and the fully connected roof state, the anti-tilt stability of a single support is stronger than the anti-slip stability, and the support is more prone to sliding instability.

**Fig. 14:**
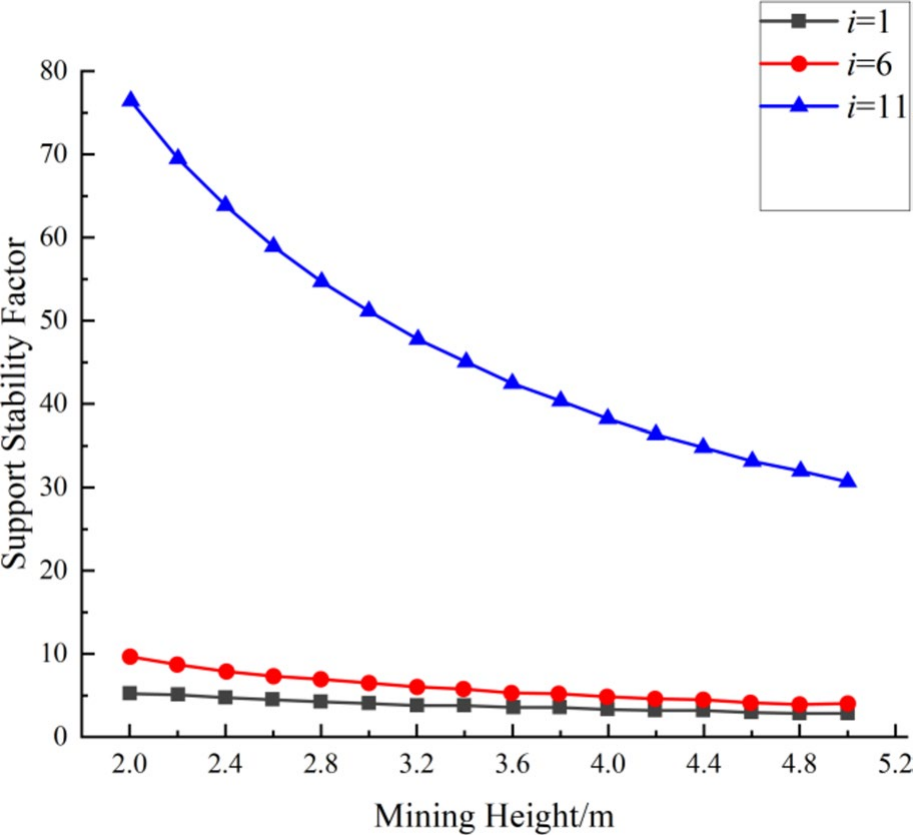
Influence of mining height on the stabilization coefficient of the support.

To explore the relationship between the anti-tilt stability coefficient of the supports at different positions of the arc section and the mining height, the upper support of the arc section (*i* = 1), the middle support of the arc section (*i* = 6), and the lower support of the arc section (*i* = 11) were selected. It was assumed that the pressure of the roof on the support was numerically equal to the self-weight of the support. The relevant parameters were substituted into Eq. (34). The relationship between the anti-tilting stability coefficients of the upper, middle and lower supports of the arc section and the mining height is shown in Fig. 14 With the increase of the mining height, the anti-tilting stability of the support gradually decreases. The lower support of the arc section has stronger anti-tilting stability than the middle and upper supports of the arc section.

Similarly, it can be concluded that the number of empty top supports above a certain support on the inclined straight section is *j*, and when the support below the support does not exert any thrust on it, the critical anti-tilting working resistance of the support can be expressed as:

36 $$P=\frac{{2G\sin \alpha h+2jH\left( {G\sin \alpha - {\mu _2}G\cos \alpha } \right) - G\cos \alpha B}}{{2\left( {{\mu _1}H+B} \right)}}$$

**Fig. 15:**
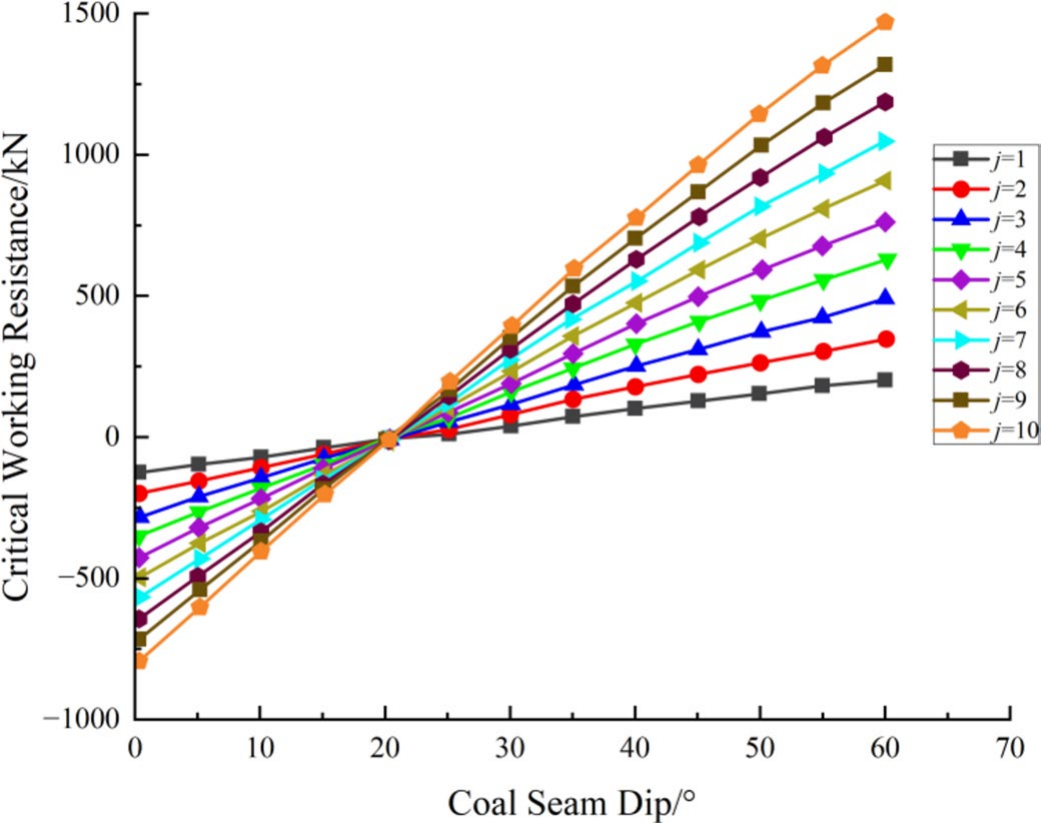
Influence of coal seam inclination on the critical anti-dumping. work resistance of the support.

Let the number of overhead supports *j* be 1 to 10. The relationship between the critical working resistance of the supports and the inclination angle of the coal seam is shown in Fig. 15.

It can be known from the analysis of Fig. 15 that when the support maintains the tilting stability by itself, the inclination angle of the coal seam increases with the increase of the number of overhead supports. With the increase of the coal seam inclination angle, the critical anti-sliding working resistance of the support increases linearly. When the inclination angle of the coal seam is constant, the greater the number of open-top supports above the supports, the greater the anti-tilt working resistance required for the supports to maintain stability.

### Analysis of the anti-tilt stability of the overall support system

During the mining process, the supports are not only closely related to the top and bottom plates, but also the interaction between the supports has a significant impact on the stability of the supports. When a certain support becomes unstable, the squeezing force *Ts* exerted by the upper adjacent support on that support is 0. When the support topples over, it will exert a squeezing force on the lower adjacent support. For the same support, *Ts ≠ Tx*, and the squeezing force between adjacent supports is equal, that is, *T*_si_=*T*_x(i−1)_,(*i* = 1 ~ *n)*.

(1) Taking the first support at the upper end of the inclined straight section as the research object, considering the bottom plate as a rigid body, and taking the critical state of the support as the condition for the support to tip over and become unstable, when the top connection is sufficient, the lateral force exerted by the first support on the second support is:

37 $${T_{hs2}}=\frac{{2G\sin \alpha h - 2PB - G\sin \alpha B}}{{2H}} - {\mu _1}P$$

The lateral force acting on the third support is:

38 $${T_{hs3}}=2\left( {\frac{{2G\sin \alpha h - 2PB - G\sin \alpha B}}{{2H}} - {\mu _1}P} \right)$$

Similarly, the lateral force exerted on the (i + 1) th support is:

39 $${T_{hs\left( {i+1} \right)}}=i\left( {\frac{{2G\sin \alpha h - 2PB - G\sin \alpha B}}{{2H}} - {\mu _1}P} \right)$$

(2) Taking the first support at the upper end of the arc section as the research object, considering the bottom plate as a rigid body, and taking the critical state of the support as the condition for the instability of the support, when the top connection is sufficient, the lateral force exerted by the first support on the second support is:

40 $${T_{qs2}}=\frac{{Gh\sin \left( {\alpha - \beta } \right)}}{{H\cos \beta }} - \frac{{{\mu _1}P}}{{\cos \beta }} - \frac{{PB}}{{H\cos \beta }} - \frac{{GB\cos \left( {\alpha - \beta } \right)}}{{2H\cos \beta }} - \frac{B}{{2H}}{T_{x1}}\tan \beta$$

The lateral force exerted by the second support on the third support is:

41 $$\begin{gathered} {T_{qs3}}=\frac{{Gh}}{{H\cos \beta }}\left[ {\sin \left( {\alpha - \beta } \right)+\sin \left( {\alpha - 2\beta } \right)} \right] - \frac{{2{\mu _1}P}}{{\cos \beta }} - \frac{{2PB}}{{H\cos \beta }} - \\ \frac{B}{{2H}}\left( {{T_{x1}}+{T_{x2}}+{T_{s2}}} \right)\tan \beta - \frac{{GB}}{{2H\cos \beta }}\left[ {\cos \left( {\alpha - \beta } \right)+\cos \left( {\alpha - 2\beta } \right)} \right] \\ \end{gathered}$$

Similarly, the lateral force exerted by the i-th support on the (i + 1) th support is:

42 $$\begin{gathered} {T_{qs\left( {i+1} \right)}}=\frac{{Gh}}{{H\cos \beta }}\left[ {\sin \left( {\alpha - \beta } \right)+\sin \left( {\alpha - 2\beta } \right)+ \cdot \cdot \cdot \cdot \cdot \cdot +\sin \left( {\alpha - i\beta } \right)} \right] - \\ \frac{{GB}}{{2H\cos \beta }}\left[ {\cos \left( {\alpha - \beta } \right)+\cos \left( {\alpha - 2\beta } \right)+ \cdot \cdot \cdot \cdot \cdot \cdot +\cos \left( {\alpha - i\beta } \right)} \right] - \\ \frac{{i{\mu _1}P}}{{\cos \beta }} - \frac{{iPB}}{{H\cos \beta }} - \frac{B}{{2H}}\left( {\sum\limits_{{n=1}}^{i} {{T_{xn}}+\sum\limits_{{n=2}}^{i} {{T_{sn}}} } } \right)\tan \beta \\ \end{gathered}$$

The compressive force exerted by the upper side support on the first support on the arc section is the compressive force exerted by the first support on the arc section on the last support of the inclined straight section. From this, the mechanical relationship of the pressure exerted by the roof on the *i*-th support on the arc section can be obtained as:

43 $$\begin{gathered} {T_{s1}}\cos \beta H+G\left[ {\sin \left( {\alpha - \beta } \right)+\sin \left( {\alpha - 2\beta } \right)+ \cdot \cdot \cdot +\sin \left( {\alpha - i\beta } \right)} \right]H= \hfill \\ \left( {{T_{x1}}+2{T_{x2}}+ \cdot \cdot \cdot +2{T_{x\left( {i - 1} \right)}}+{T_{xi}}} \right)\sin \beta \frac{B}{2}+i{\mu _1}PH+{T_{xi}}\cos \beta H+ \hfill \\ G\left[ {\cos \left( {\alpha - \beta } \right)+\cos \left( {\alpha - 2\beta } \right)+ \cdot \cdot \cdot +\cos \left( {\alpha - i\beta } \right)} \right]\frac{B}{2} \hfill \\ \end{gathered}$$

It can be known from Equations (37) to (43) that the lateral extrusion force acting on a certain support in the inclined straight section and the arc section is the superposition of the lateral extrusion force of the upper support. The inclination angle of the inclined straight section support remains unchanged, and both the lateral extrusion force and the roof pressure it receives show a linear increasing trend. The inclination angle of the arc section support gradually decreases from top to bottom. According to the trigonometric function relationship within the inclination angle range, it can be known that the lateral force acting on the arc section support and the roof pressure decrease in a parabolic linear manner. The arrangement of the arc sections improves the stress state of the lower support of the working face and reduces the risk of continuous toppling and instability of the support.

**Fig. 16:**
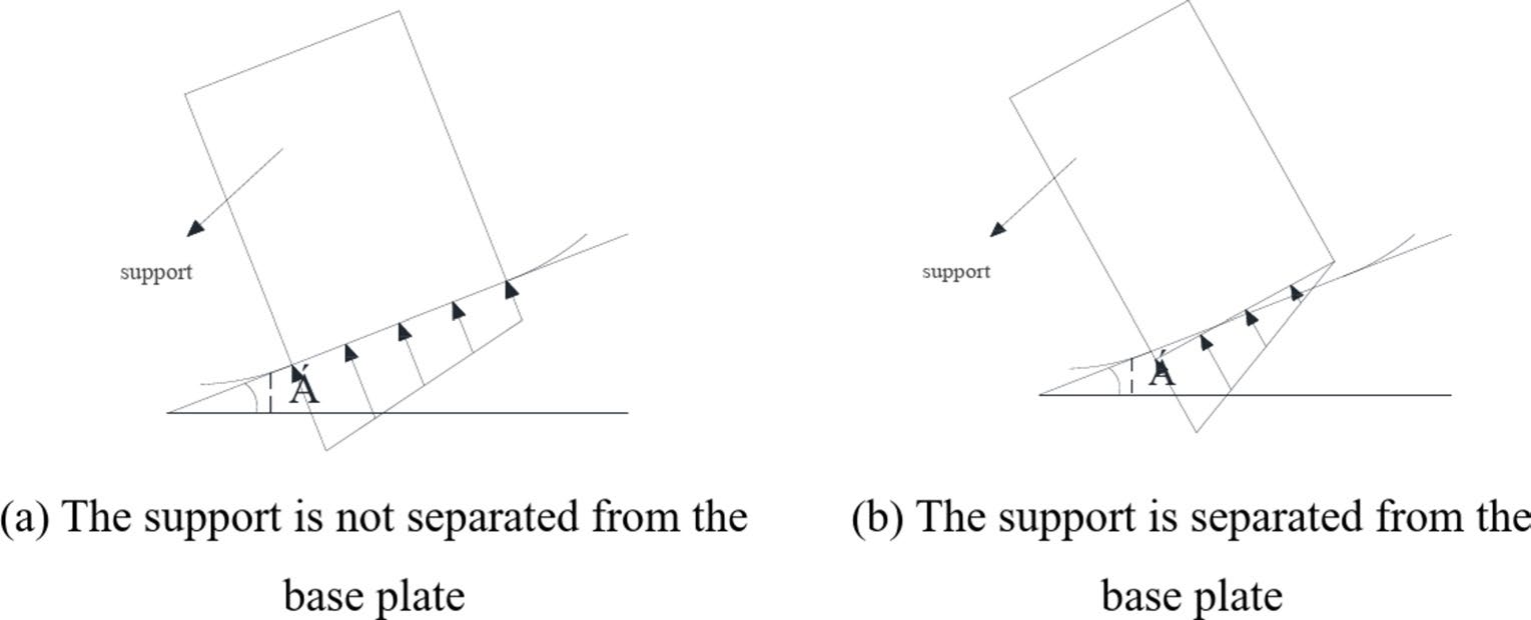
Load form for hydraulic support separated from base plate.

During the coal seam mining process, the “roof - support - floor” system of the working face is always closely connected. When the roof of the working face moves, the roof load characteristics on the support change, and the relationship between the support and the floor also changes accordingly. Assuming the base plate is an elastic foundation, the load forms of the base plate on the support can be roughly divided into uniform load, trapezoidal load and triangular load. Taking the arc section as an example, the support sinks and rotates around point *O* at the lower end of the base. The load form in which point *A* at the upper end of the support base separates from the base plate is shown in Fig. 16.

According to the elastic foundation theory, it can be calculated that the normal load resultant force R of the base plate on the support and the action position X2 are respectively:

44 $$R=\frac{{{K_o}c{Z^2}}}{{2\tan \theta }}$$

45 $${X_2}=\frac{Z}{{3\tan \theta }}$$

In the formula, *K*_o_- Elastic compression coefficient of the base plate foundation (kN/m^3^);

*Z*- Support settlement (m);

*c*- Length of the support base (m).

*θ*- Rotation angle of the support (°).

Substituting Eqs. (44) and (45) into Eqs. (27) and (28), the settlement amount *Z* of the inclined straight section support and the arc section support, as well as the tilt angle *θ* of the support can be obtained.

(1)Support for inclined straight section:

46 $$Z=\frac{{4{{\left( {P+G\cos \alpha } \right)}^2}}}{{3{K_o}b\left[ {2{f_1}H+2\left( {{T_x} - {T_s}} \right)\cos \beta H+2P{X_1}+G\cos \alpha B - 2G\sin \alpha h} \right]}}$$

47 $$\theta =\arctan \frac{{8{{\left[ {P+G\cos \alpha } \right]}^3}}}{{9{K_o}b{{\left[ {2{f_1}H+2\left( {{T_x} - {T_s}} \right)\cos \beta H+2P{X_1}+G\cos \alpha B - 2G\sin \alpha h} \right]}^2}}}$$

(2)Support for arc section:

48 $$Z=\frac{{4{{\left[ {P+G\cos \left( {\alpha - i\beta } \right)+\left( {{T_x}+{T_s}} \right)\sin \beta } \right]}^2}}}{{3{K_o}b\left[ \begin{gathered} 2{f_1}H+2\left( {{T_x} - {T_s}} \right)\cos \beta H+2P{X_1}+\left( {{T_x}+{T_s}} \right)\sin \beta B+ \hfill \\ G\cos \left( {\alpha - i\beta } \right)B - 2G\sin \left( {\alpha - i\beta } \right)h \hfill \\ \end{gathered} \right]}}$$

49 $$\theta =\arctan \frac{{8{{\left[ {P+G\cos \left( {\alpha - i\beta } \right)+\left( {{T_x}+{T_s}} \right)\sin \beta } \right]}^3}}}{{9{K_o}b{{\left[ \begin{gathered} 2{f_1}H+2\left( {{T_x} - {T_s}} \right)\cos \beta H+2P{X_1}+\left( {{T_x}+{T_s}} \right)\sin \beta B \hfill \\ +G\cos \left( {\alpha - i\beta } \right)B - 2G\sin \left( {\alpha - i\beta } \right)h \hfill \\ \end{gathered} \right]}^2}}}$$

Relevant studies have shown that the probability of sliding instability of supports in large-inclination working faces is greater than that of toppling instability. According to the parameters taken from each physical quantity in Eq. (49), it can be known that the coal seam inclination angle has a relatively small influence on the rotation angle of the support, while the mining height has a relatively large influence on the rotation angle of the support. For large inclination angles, the rotation angle of the arc section support in the general mining height working face decreases with the reduction of the support inclination angle, and the variation range is relatively small. Therefore, the probability of sliding instability of the arc section support is still greater than that of toppling instability. Corresponding measures should be taken to ensure that the support remains sliding stable.

### Quantitative analysis of the influence of arc section optimization on anti-overturning stability

Based on the anti-tipping stability coefficient *K*_q_ defined in Eqs. (34) and (35), calculate the anti-tipping capacity of the two sections of the support:

The anti-tilt coefficient of the support for inclined straight sections *K*_*q, straight*_=0.89, is in a metastable state;

The anti-tilt coefficient of the upper bracket of the arc-shaped section *K*_*q, arc−upper*_=0.94, an increase of 5.6% compared to the straight line segment;

The anti-tilt coefficient of the middle support in the arc-shaped section *K*_*q, arc−middle*_=1.32, which is 48.3% higher than the straight line segment;

The anti-tilt coefficient of the lower end support of the arc-shaped section *K*_*q, arc−lower*_=1.75, which is 96.6% higher than the straight line segment.

Similar to the anti-slip stability, the anti-toppling stability also improves significantly as the tilt Angle decreases. It is worth noting that in the inclined straight section, the anti-slip coefficient *K*_*h*_ is lower than the anti-slip coefficient *K*_*q*_, indicating that this section is dominated by sliding instability. In the middle and lower parts of the arc-shaped section, both stability coefficients are greater than 1, and the support is in a stable state.

## Distribution for field monitoring of supports characteristics

**Fig. 17:**
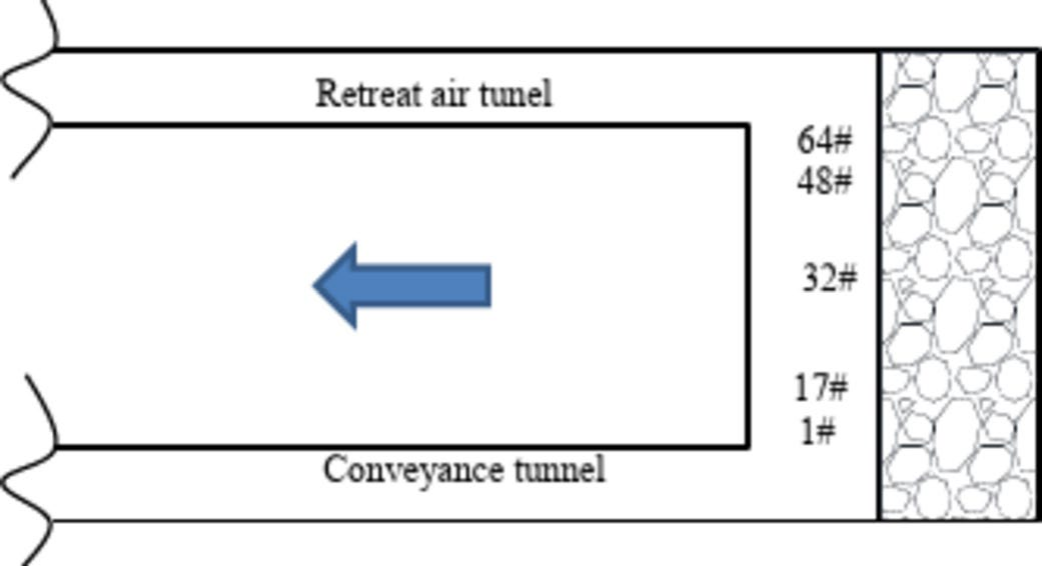
Arrangement of mine pressure monitoring stations in the working face.

The face was sectorized into upper/middle/lower dip-directional zones. Working resistance was recorded via pressure sensors on designated supports (Upper: 1#, 17#; Middle: 32#; Lower: 48#, 64#). Monitoring station arrangement appears in Fig. 17.

**Fig. 18:**
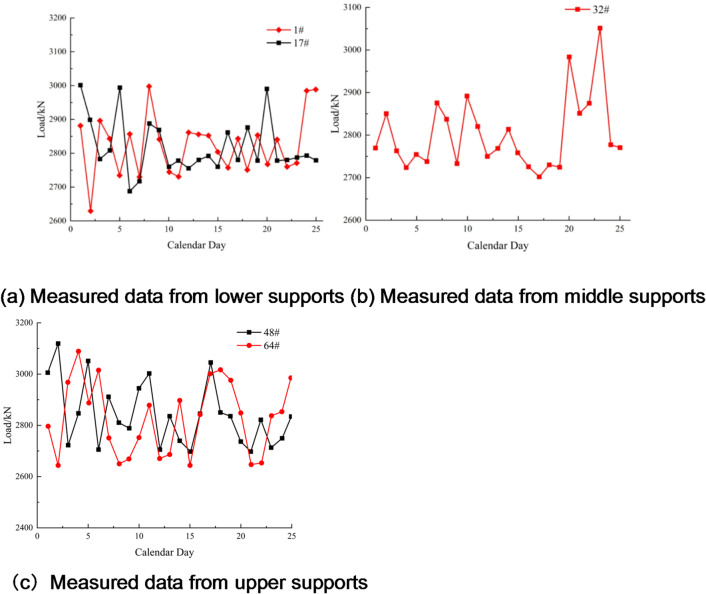
Bracket loads in the three survey areas of the working face. (As shown in Fig. 18, the 25-day monitoring record captures four to five distinct periodic weighting cycles, confirming that the observation period is sufficient to represent steady-state strata behavior at Face 32213.)

The support load distribution along the dip direction (Fig. 18), derived from tri-zone monitoring data, demonstrates varying peak resistance values: 3119kN (upper), 2894kN(middle), and 3004kN(lower). The inclination of the coal seam creates an asymmetric goaf filling pattern, resulting in phased roof weighting. In the lower face section along the dip direction, compact waste rock filling restricts strata movement, producing reduced pressure manifestations. The central dip-directional zone exhibits heterogeneous waste rock compaction, creating expanded roof fracture apertures and consequently elevated abutment pressure. The upper dip-directional zone exhibits maximal roof fracture apertures. Catastrophic buckling of the mining-induced inclined voussoir beam generates substantial dynamic stresses, producing peak abutment pressure at the face crest.

Based on the on-site monitoring data, compare the load characteristics of the inclined straight sections (Supports No. 1 and No. 17) and the curved sections (supports No. 48 and No. 64).

Table 1 Load characteristic analysis.

**Table:** 

Indicator	Inclined straight line segment	Arc section	Range of change
Average working resistance /kN	3012	2947	−
Coefficient of drag variation	0.24	0.16	Decrease by 33.3%
Exceed the rated resistance frequency	12.5%	6.2%	Reduced by 50.4%
Insufficient resistance frequency	18.3%	9.1%	Decrease by 50.3%

According to Table 1, the coefficient of variation of the support resistance in the arc-shaped section is 33.3% lower than that in the inclined straight section. The frequencies of excess resistance and insufficient resistance are both reduced by approximately 50%, indicating that the arc-shaped arrangement significantly improves the uniformity of the support load distribution along the working surface. This improvement is mainly attributed to the more balanced gangue filling in the goaf of the arc-shaped section and the more coordinated roof activities.

## Conclusion

This study investigates the mechanical stability of support structures in a steeply dipping longwall face (36°) with a curved lower-section layout. Based on theoretical modeling and field monitoring at Face 32,213, the following conclusions are drawn.

(1) Critical stability thresholds and dominant failure modes. For an individual support, the critical working resistance required to prevent sliding is greater than that required to prevent toppling. Under unsupported roof conditions, the support is more prone to sliding instability (critical sliding angle ≈ 20°) than to toppling (critical toppling angle ≈ 26.5°). Anti-sliding measures, such as increasing base-to-floor friction and installing anti-slip jacks, should therefore be prioritized in steeply dipping faces.

(2) Quantified benefits of curved-section geometry. The curved layout progressively reduces the effective inclination angle along the face, resulting in a non-linear improvement in stability. Compared with the inclined straight Sect.  (36° dip), the anti-sliding coefficient in the curved section increases from 0.82 at the upper end to 1.68 at the lower end, representing a maximum increase of 121%. The anti-toppling coefficient similarly increases from 0.94 to 1.75, representing a 96.6% improvement. The stability coefficients of the lower supports in the curved section are all greater than 1, indicating stable conditions.

(3) Load distribution uniformity and field validation. Field monitoring confirms that the curved layout significantly improves the uniformity of support loading. In the curved section, the coefficient of variation of working resistance decreased by 33.3%, while the frequencies of both over-rated and under-rated resistance occurrences were reduced by approximately 50% compared with the inclined straight section. This improvement is attributed to more uniform gangue filling and coordinated roof movement in the goaf behind the curved face.

(4) Practical guidance for support design and operation. The derived mechanical models enable quantitative prediction of critical working resistance, sliding angles, and support settlement and tilt angles. To enhance stability, it is recommended to increase the support base width and lower the center of gravity during equipment selection, as well as to expedite support advance in the unsupported-roof zone of the upper face. The curved-section layout is effective in mitigating support instability and is recommended for application in steeply dipping longwall faces under similar geological conditions.

## Data Availability

The datasets used and analyzed during the current study available from the corresponding author on reasonable request.
